# Contributions of Digital Simulation to Orthodontic Therapeutic Decision-Making: A Case Report

**DOI:** 10.7759/cureus.55540

**Published:** 2024-03-05

**Authors:** Afaf Houb-Dine, Hajar Benmohimd, Fatima Zaoui, Yassir Sabri, Asmae Bahoum

**Affiliations:** 1 Dentofacial Orthopedics Department, Faculty of Dental Medicine of Rabat, Mohammed V University, Rabat, MAR

**Keywords:** digital simulation, customized orthodontic treatment, extraction of lower incisor, class iii malocclusion, adult orthodontics

## Abstract

Adult orthodontics aims to achieve optimal functional and aesthetic corrections. However, for several reasons, some patients will wish to benefit from a limited treatment where only certain aspects of a malocclusion will be corrected. In these clinical situations, the therapeutic objectives must be adjusted to the individual needs of the patient insofar as they can bring them real benefits.

The use of digital technology makes it possible to study the therapeutic possibilities better and visualize the occlusal results before choosing the best therapeutic approach, especially in cases requiring customization.

The aim of this clinical case report is to illustrate the orthodontic compromise made after the analysis of the digital setup in an adult patient who presented with a class III malocclusion but refused orthodontic-surgical therapy and requested an alternative treatment.

## Introduction

Orthodontic-surgical therapy is indicated in most skeletal class III cases to improve occlusion and facial aesthetics. However, when faced with a refusal to undergo surgery, the orthodontist is compelled to propose a customized treatment, which may seem in certain clinical situations an interesting solution, less expensive than the global treatment with a considerably reduced treatment duration.

However, it must be remembered that this approach is an orthodontic camouflage treatment calling on dentoalveolar compensations which cannot replace an orthodontic-surgical treatment and there are underlying conditions making this compromise treatment viable.

Nowadays, digital technology has contributed to improving and simplifying treatment planning and predictability. Virtual setup is one of the aspects of this technology, representing a valuable diagnostic tool, that can be used to confirm, modify, or reject a suggested treatment plan [1].

This case report describes a customized orthodontic treatment with mandibular incisor extraction developed using digital simulation to visualize and implement the best therapeutic choice for the patient.

## Case presentation

**Figure 1:**
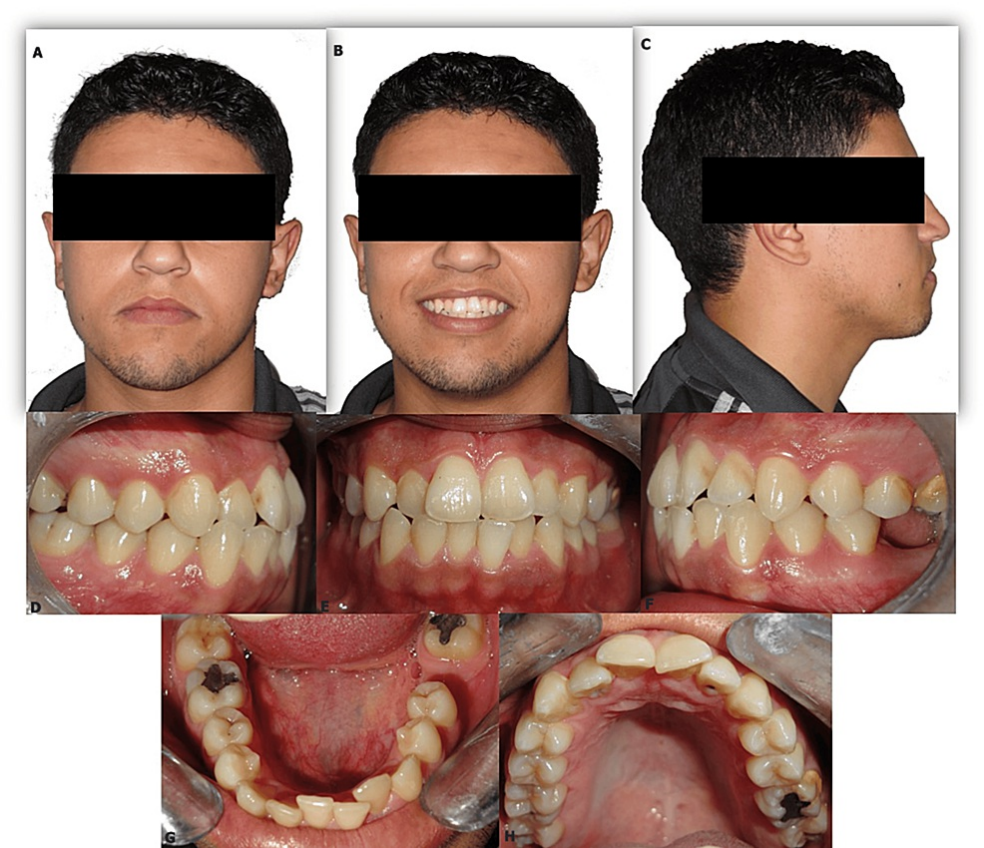
Pre-treatment extraoral (A-C) and intraoral photographs (D-H)

The patient, a 22-year-old man consulting for an aesthetic reason related to dental crowding, had a long and symmetric face with a straight profile and an open nasolabial angle. Intraoral examination revealed an Angle class I molar relationship with a history of first molar extraction in the left mandibular quadrant (i.e. 36) for endodontic reasons, an edge-to-edge incisor occlusion with a tendency to open bite, and moderate dental crowding in both arches localized at the upper and lower anterior level (Figure 1).

**Figure 2:**
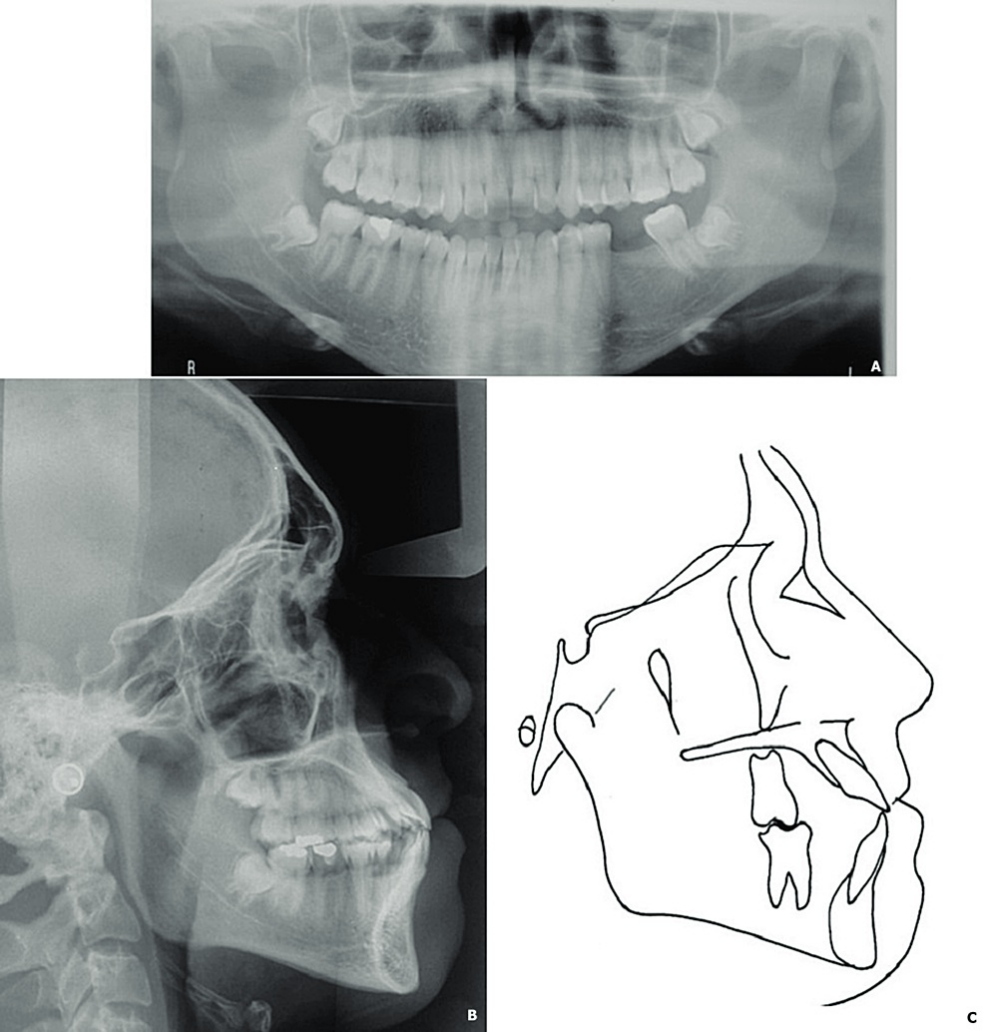
Initial radiological examination (A: panoramic radiograph; B: profile teleradiograph) and cephalometric tracing (C)

Lateral cephalometric analysis indicated a skeletal Class III relationship masked by an open mandibular plane angle as well as protrusion of the upper and lower incisor (Figure 2, Table 1).

**Table 1:** Initial cephalometric values SNA: the maxilla (point A) is related to the cranial base (SN) SNB: the mandible (point B) is related to the cranial base (SN) ANB: is the difference between SNA and SNB SND: the point D is the center of the symphysis of the mandible I/to NA (mm): maxillary incisor position I/to NA: maxillary incisor version i/ to NB (mm): lower incisor position i/ to NB: lower incisor version Po to NB: most anterior part of the symphysis of the mandible to line NB I / to / i: interincisal angle Occl to SN: occlusal plane to the cranial base GoGnSN: mandibular plane to cranial base SL: represents the body of the mandible SE: represents the implantation of the mandible to the cranial base FMA: Frankfort mandibular plane angle IMPA: the lower incisor's axial inclination to the mandibular plane AoBo: represents the projection of the points A and B on the occlusal plane

Parameters	Normal values	Initial patient values
SNA	82	81
SNB	80	80
ANB	2	1
SND	76	76
I/to NA	4	9
I/to NA	24	32
i/ to NB	4	10
i/ to NB	24	33
Po to NB		0
I / to / i	131	113
Occl to SN	14	15
GoGnSN	32	39
SL	51	44
SE	22	14
FMA	25	27
IMPA		95
AoBo	0	-4

Treatment plan and procedure

In the present case, the ideal therapy would be an orthodontic-surgical treatment with a genioplasty for vertical reduction and anterior transposition to reduce the anterior vertical excess and medialization for 37 and 38, however, the patient had declined this option and insisted on an alternative approach without surgery and a short duration of appliance wear.

**Figure 3:**
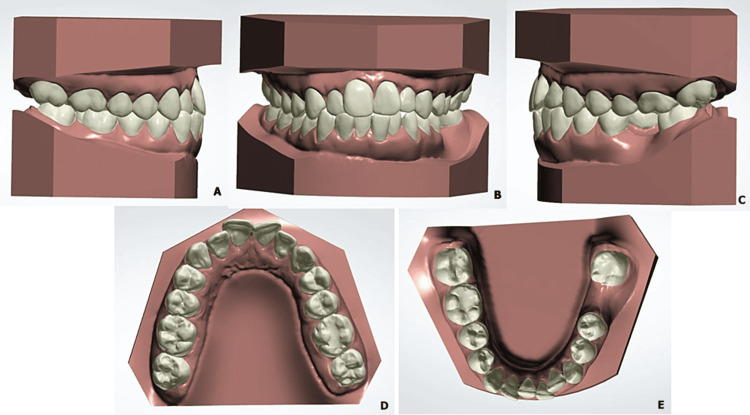
Pre-treatment digital casts Pre-treatment digital casts (views: A: right side, B: anterior view, C: left side, D: maxillary arch, E: lower arch)

Taking into account the flat profile of the patient, we proposed an orthodontic treatment by compensation and extraction of a lower incisor. Before undergoing treatment, we developed a digital simulation from digital casts to discuss this option (Figure 3).

For that, the initial plaster model was scanned with a desktop scanner in order to obtain a digital model. On this digital model, a virtual setup was made with 3Shape Ortho System version 19.1 (3Shape, Copenhagen, Denmark). 

**Figure 4:**
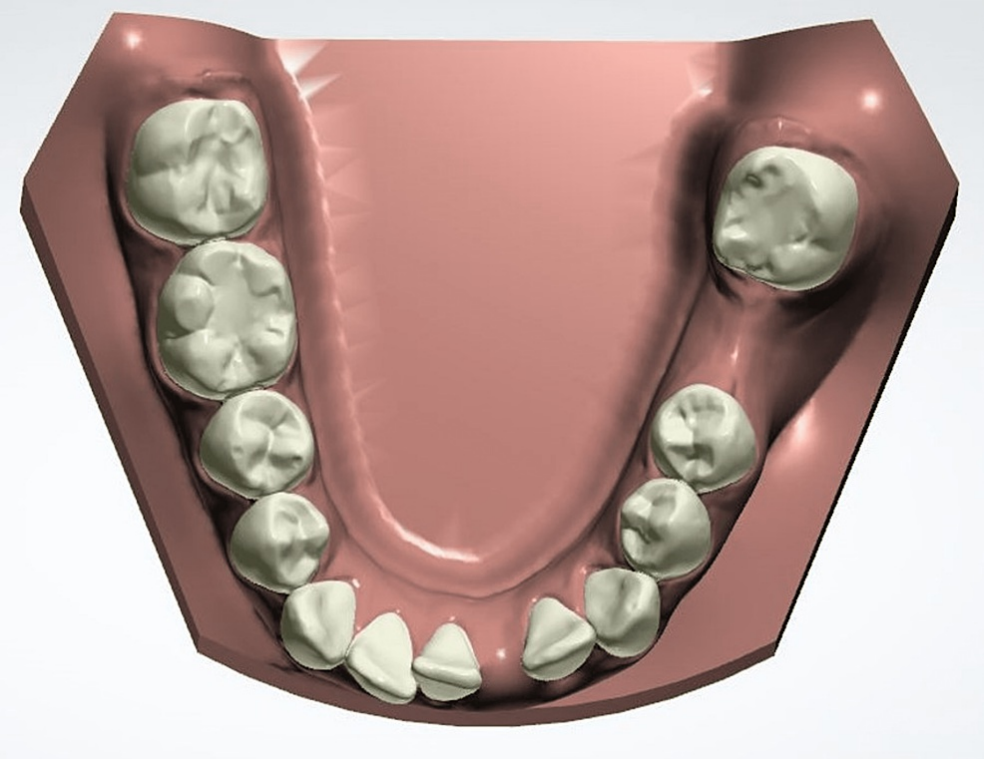
Mandibular virtual cast after extraction of 31

**Figure 5:**
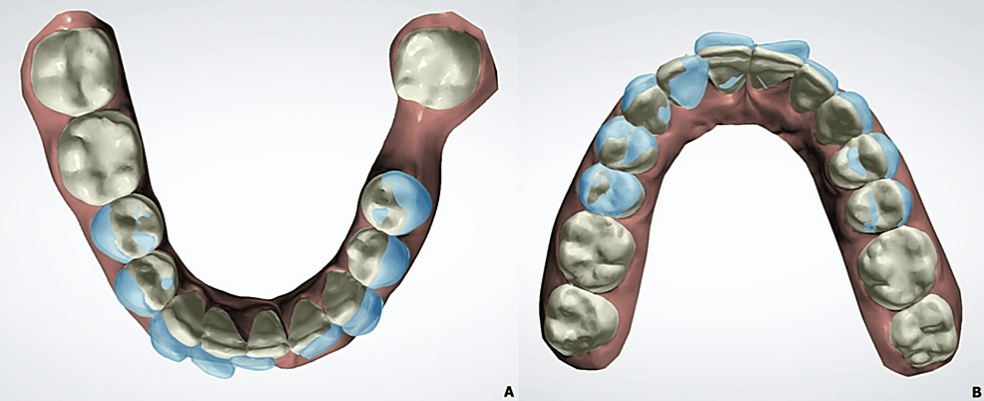
Superimposition of digital models before (A) and after simulation (B)

**Figure 6:**
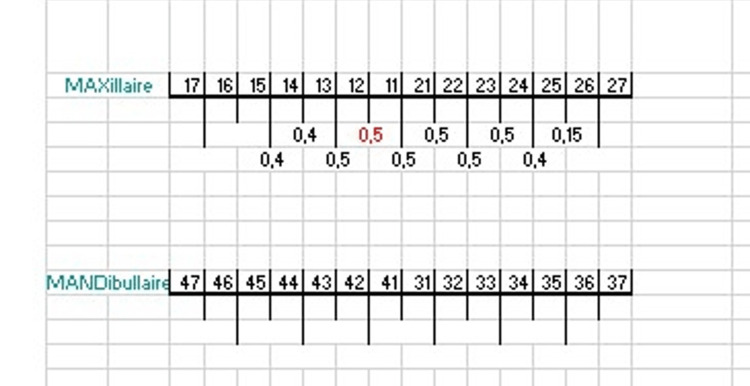
Maxillary stripping guide (location and quantity) Maxillaire: maxillary arch; mandibulaire: mandibular arch

The digital simulation allowed us to visualize the occlusion after extraction of 31 (Figure 4), to determine the amount and direction of movement for each tooth (Figure 5), and to evaluate the need for stripping of the maxillary incisors before validating our therapeutic project (Figure 6).

**Figure 7:**
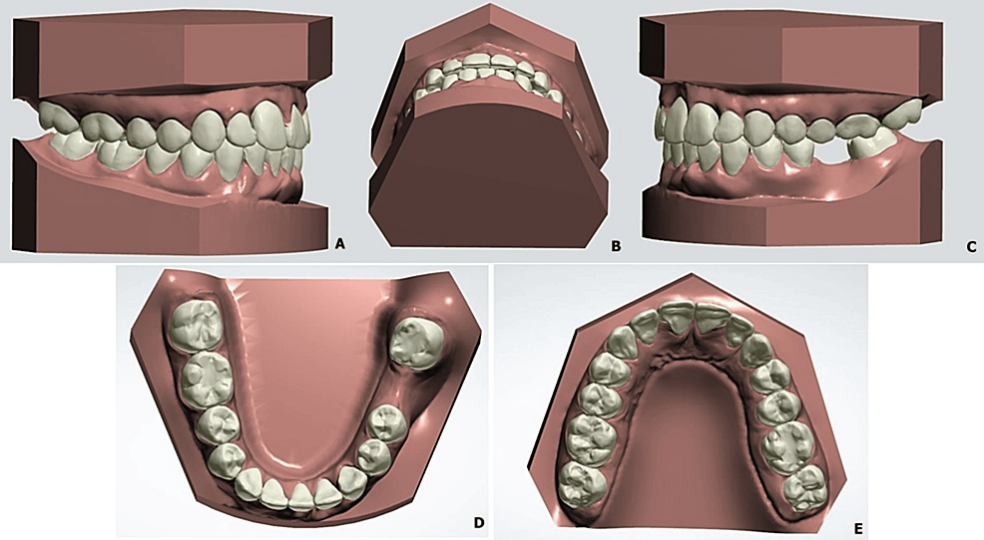
Virtual setup simulation of post-treatment occlusion after extraction of a lower incisor Views: A, right side; B, lower occlusion; C, left side; D, lower arch; E, maxillary arch)

The setup results showed the alignment of the anterior teeth while maintaining at the lateral level a class I occlusal relationship. The use of stripping in the maxilla made it possible to preserve the incisor's periodontium and to compensate for the tooth size discrepancy created by the extraction of the lower incisor (Figure 7).

We showed this provisional model to our patient who gave his consent for this compromise treatment with extraction of 31, stripping of the upper maxillary incisors, extraction of wisdom teeth, and implant to replace 36 later.

Treatment results

**Figure 8:**
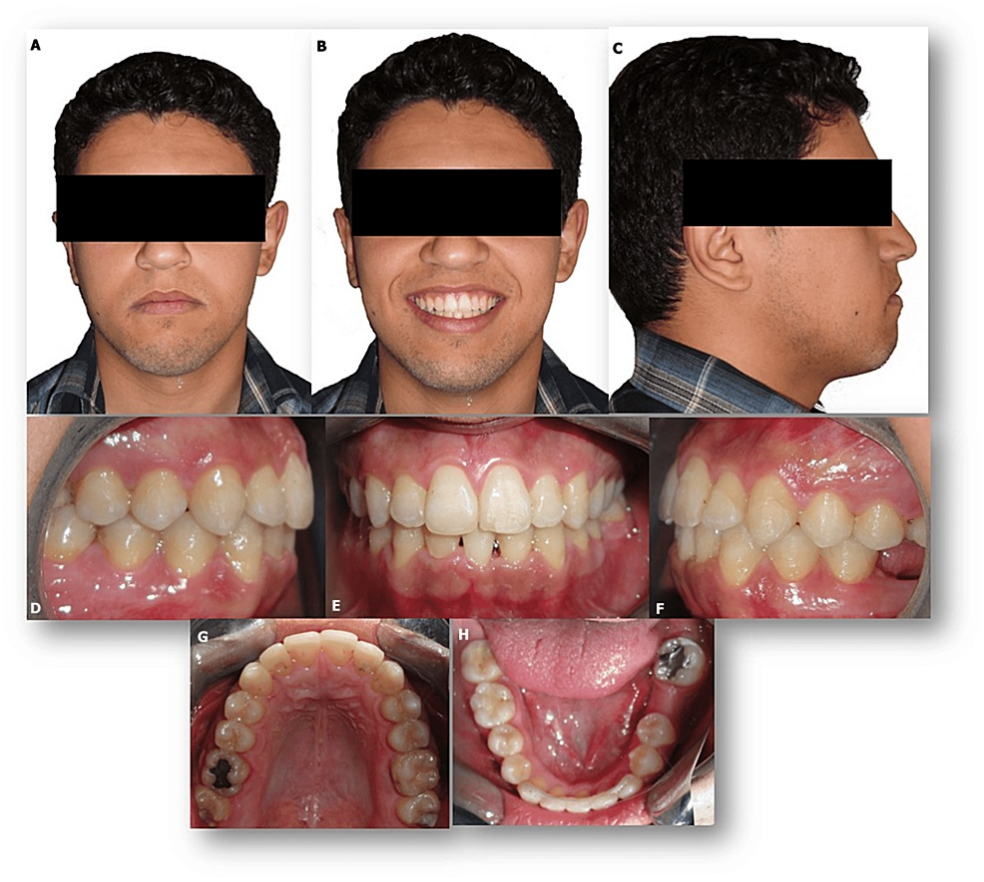
Post-treatment extraoral (A-C) and intraoral photographs (D-H)

Orthodontic treatment lasted approximately 18 months. At the end of the full-fixed treatment, crowding was eliminated, overjet and overbite were restored and the Class I canine and molar relationship was preserved. The median upper incisor coincided with the middle of the 41. The stabilization phase was carried out with a 3-3 bonded retainer and a thermoformed jaw splint (Figure 8).

**Figure 9:**
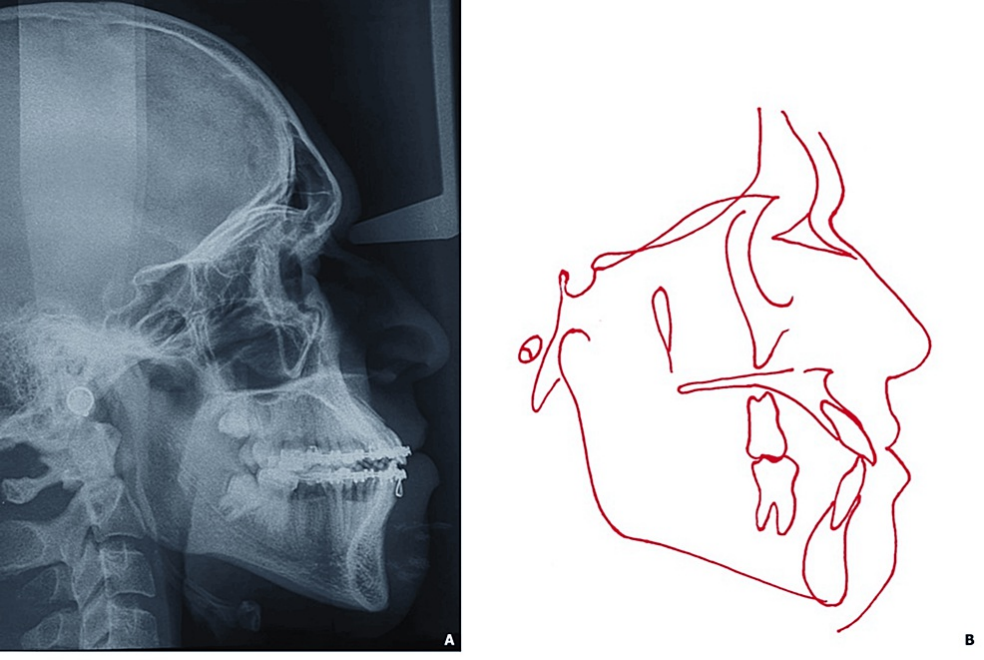
Post-treatment profile teleradiograph (A) and cephalometric tracing (B)

**Figure 10:**
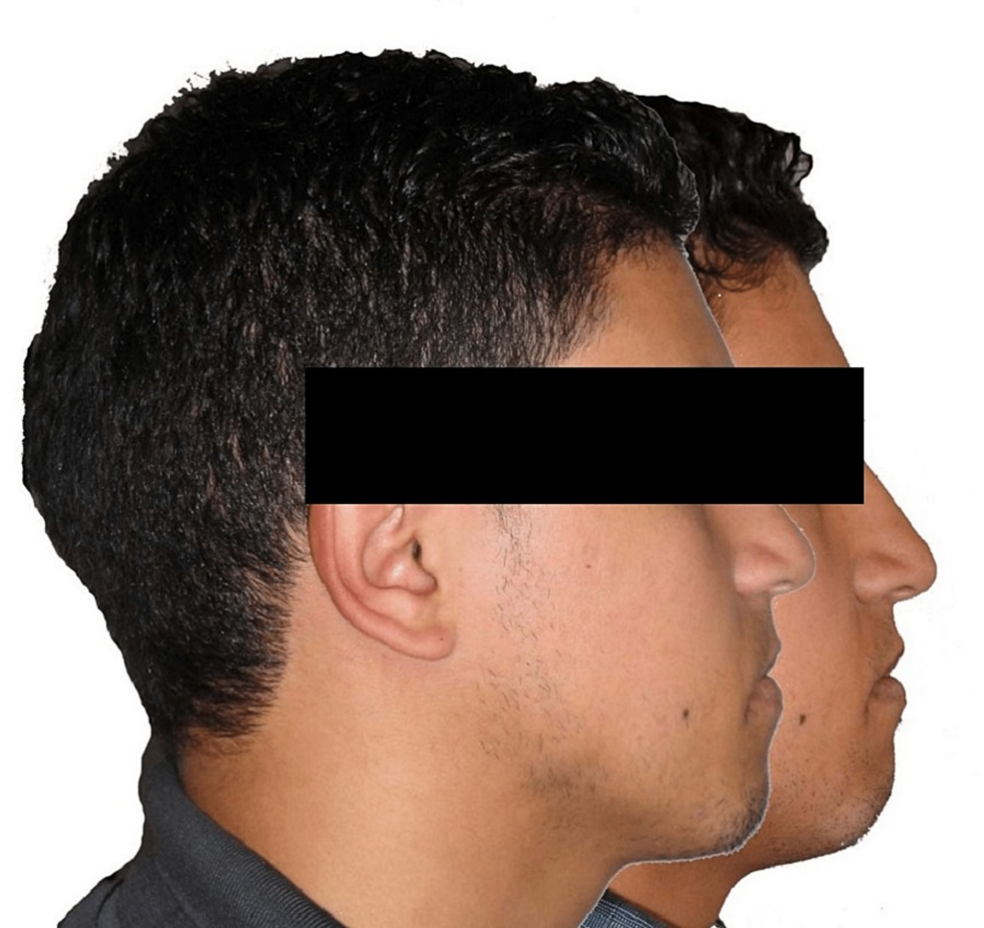
Profile superimposition

Post-treatment cephalometric analysis and superimposition revealed no changes of the profile (Figures 9-10; Table 2).

**Table 2:** Post-treatment cephalometric analysis SNA: the maxilla (point A) is related to the cranial base (SN) SNB: the mandible (point B) is related to the cranial base (SN) ANB: is the difference between SNA and SNB SND: the point D is the center of the symphysis of the mandible I/to NA (mm): maxillary incisor position I/to NA: maxillary incisor version i/ to NB (mm): lower incisor position i/ to NB: lower incisor version Po to NB: most anterior part of the symphysis of the mandible to line NB I / to / i: interincisal angle Occl to SN: occlusal plane to the cranial base GoGnSN: mandibular plane to cranial base SL: represents the body of the mandible SE: represents the implantation of the mandible to the cranial base FMA: Frankfort mandibular plane angle IMPA: the lower incisor's axial inclination to the mandibular plane AoBo: represents the projection of the points A and B on the occlusal plane

Parameters	Normal values	Initial patient values	Post-treatment patient values
SNA	82	81	81
SNB	80	80	80
ANB	2	1	1
SND	76	76	76
I/to NA	4	9	10
I/to NA	24	32	35
i/ to NB	4	10	9
i/ to NB	24	33	27
Po to NB		0	0
I / to / i	131	113	118
Occl to SN	14	15	15
GoGnSN	32	39	39
SL	51	44	43
SE	22	14	14
FMA	25	27	27
IMPA		95	91
AoBo	0	-4	-5

The patient was satisfied with the final result, after placement of the retainer, the patient was first referred to the oral surgery department for the extraction of wisdom teeth and then to the prosthesis department for the replacement of tooth 36.

## Discussion

Skeletal class III malocclusion in adults is one of the most challenging dysmorphosisms to treat. It can be managed according to orthodontic-surgical protocols or simply orthodontic camouflage treatment to cover underlying skeletal discrepancies, with satisfactory results for both approaches. The choice of approach depends on the severity of the skeletal problem, growth pattern, and facial profile.

The ideal therapy for this case was the use of an orthodontic-surgical approach which remains the gold standard since it enables the attainment of better aesthetic and functional results; however; the patient had declined this orthodontic-surgical option.

Another treatment option was the extraction of the first premolars; however, this would lead to a more retrusive upper lip, which would further worsen the profile. Therefore, our remaining choice was a compromise treatment with lower incisor extraction.

The extraction of the mandibular incisor in this patient contributed favorably to the correction of the mandibular dental crowding, which was his main reason for consultation. The profile alteration was avoided. The treatment lasted 18 months, which is a short period compared to the duration of other orthodontic approaches.

Clinical results of this case matched the ones reported in the literature which underline that the extraction of a mandibular incisor could be a good solution to correct the mandibular dental crowding, especially in cases of Class I molar and canine relationship with a 4 or 5 mm of anterior crowding and Class I or Class III tendency associated with an edge-to-edge occlusion of the incisors, anterior crossbite and minimal overbite, or open bite tendency [2-4].

This unique choice of extraction is an unconventional solution since it necessarily imposes an atypical occlusion where five mandibular teeth are aligned with six maxillary teeth. This particular occlusion is constructed thanks to balanced vertical and sagittal anterior occlusal relationships in both static and dynamic occlusions [5]. This delicate decision can be previewed by carrying out a diagnostic set-up on the articulator manually or with digital technology using digital set-up in order to detect and visualize some undesirable results, namely the increase in overjet and overbite and the disturbance of lateral occlusal relationships [6, 7].

Thanks to the realization of this provisional model, we were able to visualize the final occlusion of our patient and to make our therapeutic choice with full knowledge of the facts and in consultation with the patient [8]. This digital simulation is particularly useful for borderline cases and remains the best diagnostic measure to assess the final occlusion [9].

We extracted 31; the choice of extraction fell on the left central incisor which comes out of the incisor arch, which therefore allowed us to preserve the canine class I occlusal relationships.

Several authors recommend the extraction of the medial incisor to obtain at the end an occlusion where the median upper incisor corresponds to half of the vestibular face of the remaining central lower one and thus to avoid a significant mesialization of the lower canine during the extraction of the lateral incisor, which could cause non-working interference during laterality movements [3].

Unaesthetic loss of interdental papillae in the mandibular anterior region in our patient is one of the common side effects of lower incisor extraction [8, 10, 11]. This negative aesthetic factor will be less pronounced for incisors with rectangular crown shapes [7].

Interproximal reduction could have been a promising approach to reduce their appearance, however, the enamel width wasn't propitious to apply this reduction. Moreover, an additional upper incisor reduction would have been necessary to maintain a functional overjet and overbite.

Particular attention should be paid to the finishing of these cases and their 3x3 bonded retention to avoid recurrence [2].

Keeping in mind the various factors mentioned above, the implementation of the diagnostic setup, and the practitioners' clinical and critical sense are all conditions that can lead to good results making the choice of orthodontic treatment with extraction of lower incisor an acceptable compromise.

## Conclusions

This case report shows the benefits of using a digital montage to facilitate clinical decision-making. The patient's expectations of rapid treatment were taken into account.

Compromise orthodontic treatment is an alternative treatment used instead of the overall orthodontic treatment recommended for adult patients. It should be the responsibility of the practitioner to direct their patient toward this solution after a good diagnosis. Knowledge of the treatment limits should not be exceeded and mastery of orthodontic mechanics is a must. With a digital treatment simulation process, orthodontists can plan, visualize, and predict treatment results, which contributes to better communication between orthodontists and their patients and guarantees a better choice of therapeutic approach, thus meeting patient expectations.
